# Exploring the potential of a *Ephedra alata* leaf extract: Phytochemical analysis, antioxidant activity, antibacterial properties, and green synthesis of ZnO nanoparticles for photocatalytic degradation of methylene blue

**DOI:** 10.3389/fchem.2024.1367552

**Published:** 2024-02-21

**Authors:** Abdelmalek Zaater, Mohammed Oualid Serhoud, Ilham Ben Amor, Soumeia Zeghoud, Amira Hemmami, Abdelkrim Rebiai, Yacine Bouras, Ammar Touhami Laiche, Ali Alsalme, David Cornu, Mikhael Bechelany, Ahmed Barhoum

**Affiliations:** ^1^ Biodiversity Laboratory and Application of Biotechnology in Agriculture, University of El Oued, El Oued, Algeria; ^2^ Department of Agronomy, Faculty of Nature and Life Sciences, University of El Oued, El Oued, Algeria; ^3^ Laboratory of Community and Family, University of Batna 1, Batna, Algeria; ^4^ Department of Sociology and Demography, Faculty of Humanities and Social Sciences, University of Batna 1, Batna, Algeria; ^5^ Department of Process Engineering and Petrochemical, Faculty of Technology, University of El Oued, El Oued, Algeria; ^6^ Renewable Energy Development Unit in Arid Zones (UDERZA), University of El Oued, El Oued, Algeria; ^7^ Department of Biology, Faculty of Natural Science and Life, University of El Oued, El Oued, Algeria; ^8^ Laboratory of Applied Chemistry and Environment, Faculty of Exact Sciences, University of El Oued, El Oued, Algeria; ^9^ Chemistry Department, Faculty of Exact Sciences, University of El Oued, El Oued, Algeria; ^10^ Laboratory Biology, Environment and Health (LBEH), University of El Oued, El Oued, Algeria; ^11^ Department of Chemistry, College of Science, King Saud University, Riyadh, Saudi Arabia; ^12^ Institut Européen des Membranes (IEM), UMR, University Montpellier, ENSCM, CNRS, Montpellier, France; ^13^ Gulf University for Science and Technology, GUST, Hawally, Kuwait; ^14^ NanoStruc Research Group, Chemistry Department, Faculty of Science, Helwan University, Cairo, Egypt

**Keywords:** plant extract phytochemical analysis, high-performance liquid chromatograph, antioxidant activity, ZnO nanoparticle synthesis, antibacterial activity, photocatalytic degradation, methylene blue dye degradation

## Abstract

Ephedra alata leaf extracts have therapeutic properties and contain various natural compounds known as phytochemicals. This study assessed the phytochemical content and antioxidant effects of a *Ephedra alata* leaf extract, as well as zinc oxide (ZnO) nanoparticle production. The extract contained phenolic acids, including vanillic acid, chlorogenic acid, gallic acid, p-coumaric acid, vanillin and rutin. Its total phenolic content and total flavonoid content were 48.7 ± 0.9 mg.g^-1^ and 1.7 ± 0.4 mg.g^-1^, respectively. The extract displayed a DPPH inhibition rate of 70.5%, total antioxidant activity of 49.5 ± 3.4 mg.g^-1^, and significant antimicrobial activity toward Gram-positive and negative bacteria. The synthesized ZnO nanoparticles had spherical shape, crystallite size of 25 nm, particle size between 5 and 30 nm, and bandgap energy of 3.3 eV. In specific conditions (90 min contact time, pH 7, and 25°C), these nanoparticles efficiently photodegraded 87% of methylene blue, suggesting potential applications for sustainable water treatment and pollution control.

## Graphical Abstract

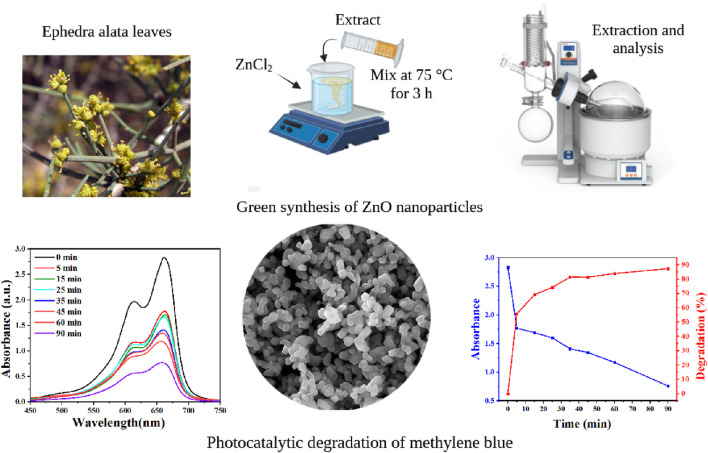

## 1 Introduction

Plant extracts are important materials in many fields, due to their versatility. As they are valuable sources of phytochemical compounds, including flavonoids, phenolic compounds, alkaloids, and tannins, they are used in traditional medicine as well as in pharmaceuticals and cosmeceuticals (Tungmunnithum et al., 2018). These compounds also display antioxidant and antibacterial activities attributed to their redox properties and unique chemical structures. Recently, plant extracts have gained attention as reducers and stabilizers to facilitate the production of metal and metal oxide nanoparticles (NPs) (Tungmunnithum et al., 2018). In this process, the plant extract is combined with metal precursors and the metal ions undergo specific steps (reduction, nucleation, growth and stabilization). This plant-based synthesis approach is eco-friendly, cost-effective, simple, and potentially scalable for industrial production (Kulkarni et al., 2023; Radulescu et al., 2023). However, it is challenging to precisely control particle size, morphology, and crystallinity. In addition, the plant extract composition variability and the different sources of such extracts add another level of complexity. Ongoing research is focusing on reaction condition optimization, procedure standardization, and understanding the role of individual phytochemicals in NP synthesis to fully exploit this method of NP production (Antunes Filho et al., 2023; Singh et al., 2023).

Various plant extracts have been tested for the synthesis of ZnO NPs with different sizes and morphologies. For instance, an *Aloe vera* leaf extract was used to produce ZnO NPs with dimensions ranging from few nanometers to micrometers (Kumar et al., 2015). ZnO NPs take a nanoflower structure when shaped using a green tea leaf extract, and display sizes within 30–40 nm (Irshad et al., 2018). Neem leaf extract facilitates the synthesis of non-spherical ZnO NPs with a size of 20 nm (Sohail et al., 2020). Turmeric, rich in curcumin, has been used to produce spherical ZnO NPs with reported sizes of ∼40 nm (Singh et al., 2016). ZnO NPs derived from an orange fruit peel extract exhibited a spherical-like morphology (30–40 nm in size) (Thi et al., 2020). Neem (*Azadirachta indica*) and tulsi (*Ocimum tenuiflorum*) extracts yielded oval-shaped ZnO NPs with sizes of 100 nm and 122 nm, respectively (Ajayan and Hebsur, 2020). A mangrove leaf extract was used to synthesize spherical ZnO NPs with a size of ∼30 nm (Al-Mur, 2023). Using *Costus pictus* D. extract, ZnO NPs with tailored size and morphology were fabricated (hexagonal and rod shapes with a reported size of 29 nm) (Suresh et al., 2018). ZnO NPs with a particle size of ∼35 nm were obtained using an aqueous extract of dried onion (*Allium cepa* L) peels (Rajkumar et al., 2019). A *Salvia rosmarinus* extract allowed the production of ZnO NPs with reported sizes of ∼31 nm and a spherical shape (Xue et al., 2023). Pure curcumin extract was used to synthesize ZnO NPs with a size of 28 nm (Alallam et al., 2023).

*Ephedra alata*, commonly known as ma-huang, is a widespread shrub found in various countries that originates from Southwestern North America, Southern Europe, and North Africa. This medicinal plant is part of the *Ephedra* genus (Elhadef et al., 2020). Previous studies highlighted its diverse biological effects: antibacterial properties, anti-inflammatory effects, cardiovascular disease prevention, and cancer prevention and treatment (Saidi et al., 2022). *Ephedra alata* leaves contain various phytochemicals, including flavonoids, alkaloids and phenolic compounds. These leaves have been used as reducing and capping agents for the synthesis of gold, silver, and copper oxide NPs (Mousavi et al., 2018; Al-Radadi, 2023; Antonio-Pérez et al., 2023; Atri et al., 2023; Bouzid et al., 2023). Therefore, *Ephedra alata* extracts could be used for NP production in order to address the concerns associated with the very high toxicity of conventional NP production systems that poses risks to human and animal health. Research suggests that *Ephedra alata* extracts possess antioxidant properties, playing a role in neutralizing free radicals to mitigate oxidative stress and cellular damage (González-Juárez et al., 2020). Moreover, the alkaloids found in *Ephedra alata*, particularly ephedrine and pseudoephedrine, have antimicrobial effects against some bacteria and fungi (González-Juárez et al., 2020; Jaradat et al., 2021). These findings indicate that *Ephedra alata* is not only a medicinal plant, but also a valuable resource for the eco-friendly synthesis of NPs for antimicrobial applications.

This study comprehensively assessed the applications of an *Ephedra alata* leaf extract by analyzing its phytochemical composition, antioxidant properties, antimicrobial effects, and its value for zinc oxide (ZnO) NP production. The extract components were identified by phytochemical and high-performance liquid chromatography (HPLC) analyses, forming the basis for understanding its biological activities. Evaluation of the extract antioxidant and antimicrobial activities, particularly against Gram-negative (−) and positive (+) bacteria, provided specific insights into its potential health benefits. The study underscored the extract eco-friendly potential for ZnO NP synthesis and assessed the capacity of these NPs to degrade an azo dye. Leaf extracts, serving as reducing and stabilizing agents, contribute to the unique NP properties. Green NPs from leaf extracts display eco-friendliness, sustainability, and versatility. The ZnO NP morphological and structural characterization explained their potential role in sustainable water treatment. The practical applications of these findings, including the development of natural antioxidants and eco-friendly dye degradation solutions, highlight their significant impact in different industries (pharmaceutical and cosmeceutical production and sustainable water treatment).

## 2 Experimental

### 2.1 Materials

Zinc chloride (ZnCl_2_, 98%), sodium hydroxide (NaOH, 97%), acetic acid (CH_3_COOH, 98%), DPPH (C_18_H_12_N_5_O_6_, 98%), ABTS (C_18_H_18_N_4_O_6_S_4_, 98.5%), sodium dihydrogen phosphate (NaH_2_PO_4_, 98%), disodium phosphate (Na_2_HPO_4_, 97%), ammonium persulfate ((NH_4_)_2_S_2_O_8_, 98%), sodium chloride (NaCl, 99%), phosphate (PO_4_^3-^, 98%), sulfuric acid (H₂SO₄, 98%), ascorbic acid (C_6_H_8_O_6_, 99.5%), aluminum molybdate (Al_2_(MoO_4_)_3_, 98%), sodium phosphate (Na_3_PO_4_, 99%), methanol (CH_3_OH, 99%), dimethyl sulfoxide (DMSO, 99%), and methylene blue (MB; C_16_H_18_ClN_3_S, 82%) were from Biochem Chemophara. Mueller-Hinton agar was from Bioscan Industrie, Algeria. *Ephedra alata* leaves were collected at El Oued, Algeria (6°52′03″E, 33°22′06″N), in March 2022. They were identified by an engineer specialized in botany. Leaf samples were stored in the dark, at room temperature, in dry and cool conditions.

### 2.2 Extraction method

The leaf extract was prepared according to the procedure described by Zeghoud et al. (Zeghoud et al., 2021), with some changes. Briefly, 20 g of *Ephedra alata* leaves were ground and added to a 500 mL Erlenmeyer flask that contained 200 mL of ethanol. The flask was placed in an ultrasonic bath (Power-Sonic, China) that operated at 40 kHz and 250 W. To counteract potential temperature increases resulting from the intense molecular agitation induced by ultrasound, the ultrasonic bath was maintained at a constant temperature (30°C) for 30 min. To protect light-sensitive molecules from degradation, the flask was wrapped in aluminum foil. After filtration using filter paper, ethanol was separated from the filtrate using a rotary evaporation apparatus (Büchi R-210) under reduced pressure. Then, the ethanol was oven-dried at a temperature ≤40°C for at least 48 h. Desiccated ethanol was transferred into an amber glass bottle and kept at −4°C.

### 2.3 Phytochemical analysis

*Alkaloid Test*: This test was used to determine the presence of alkaloids in the extract. Briefly, 10 mL of the extract of (1 mg.mL^-1^) was prepared by dissolving the appropriate amount in absolute methanol and heating it at a temperature of 45°C for 2 h. Then, 8 mL of 1% HCl solution was added. To detect alkaloids, two separate 2-mL aliquots of the filtrate were taken, and the Dragendorff’s and Mayer’s reagents were added to each aliquot. The presence of alkaloids was determined by observing the formation of turbidity and/or precipitation in the respective aliquots (Kancherla et al., 2019).

*Saponin Test:* Saponins are naturally occurring compounds with foaming properties found in various plants. In this method, 2 g of plant powder was boiled in distilled water followed by filtration to obtain a clear filtrate. Subsequently, a robust foam was generated by mixing (swiftly agitating) 10 mL of this filtrate with 5 mL of distilled water. The key step involved the creation of an emulsion by vigorously mixing the foam with three drops of extra-virgin olive oil. The formation of this emulsion serves as an indicative sign of the presence of saponins in the plant sample (Chen et al., 2022).

*Tannin Test:* In this test, ground *Ephedra alata* (0.5 g) was simmered in 20 mL of distilled water and then filtered through filter paper. Afterwards, 0.1% FeCl_3_ was added to the sample. The presence of tannins was indicated by the development of a distinctive brownish-green color in the filtered samples (Edrah et al., 2016).

*Keller-Killani Test:* This test detects the presence of cardenolide glycosides in a methanolic plant extract. Briefly, 2 mL of glacial acetic acid, 1 mL of concentrated H_2_SO₄, one drop of FeCl_3_ solution and 5 mL extract of (1 mg.mL^-1^ in methanol) were mixed. The appearance of a violet ring under the brown ring in the acetic acid layer is a distinct indicator of the presence of cardenolide glycosides. The test also allows monitoring additional color changes, such as the appearance of a violet ring beneath the brown ring in the acetic acid layer (Ahmed et al., 2020).

*Coumarin Test:* To detect the presence of coumarins, a wet 0.5 g sample of the leaf extract was placed in a test tube, which was sealed with filter paper wetted in 0.5 mL of aqueous 10wt% NaOH solution. After a brief immersion in boiling water, the filter paper was removed and exposed to ultraviolet (UV) light. The presence of coumarins was indicated by the appearance of a distinct yellow fluorescence at 700 W UV light (Kostova et al., 2011).

*Terpenoid Test:* Briefly, 2 mL of the extract (1 mg.mL^-1^ in methanol) was placed in a test tube with 2 mL of aqueous 5v/v% CHCl_3_. Then, 3 mL of concentrated H_2_SO_4_ was added to the mixture. The introduction of these reagents led to the formation of distinct layers in the test tube. The key indicator of the presence of terpenoids is the appearance of an interface with a reddish-brown hue between the layers (Awala and Oyetayo, 2015).

*Flavonoid Test - Shinoda Test:* The test is employed to detect the presence of flavonoids in the extract. The dry extract was mixed with 0.5 g of magnesium powder and 1 mL of aqueous HCl solution (11.94 mol.L^-1^). The presence of flavonoids is indicated by the rapid appearance of a distinctive pinkish-scarlet hue in the mixture (Auwal et al., 2014).

*Volatile Oil Test:* This test is used to identify oils in plant extracts. Briefly, 1 mL sample of the extract (1 mg.mL^-1^ in methanol) was combined with 2 mL of 50wt% KOH solution. The presence of volatile oils is indicated by the rapid appearance of distinct needle-shaped crystals in the mixture (Djaafar and Ridha, 2014).

*Total Phenolic Content (TPC) Determination:* TPC in the *Ephedra alata* leaf extract was determined using Folin-Ciocalteu reagents and the method described by Beretta et al. (Jaradat et al., 2015; Lawag et al., 2023). The crude extract (500 μL of a 1 mg.mL^-1^ in water) and 0.25 mL of Folin-Ciocalteu phenol reagent were combined and after 3 min, 1 mL of aqueous 7.5wt% Na_2_CO_3_ was added. After 30 min in the dark, absorbance at 760 nm was measured using a Jasco V160 UV-Vis spectrophotometer. TPC was determined using a calibration curve made with gallic acid as standard (0.01–0.0375 g.L^-1^ in distilled water) and reported as mg of gallic acid equivalents (GAE) (mg.kg^-1^).

*Total Flavonoid Content (TFC) Determination:* TFC was assessed using the AlCl_3_ colorimetric test (Shraim et al., 2021). A solution was prepared using 1,250 μL of distilled water, 75 μL of aqueous 5wt% NaNO_2_, and 50 μL of diluted plant extract (1 mg.mL^-1^ in methanol) or standard catechin solution (0.2–1 mg.mL^-1^ in distilled water). After 6 min, 150 μL of fresh aqueous 10wt% AlCl_3_ solution was added. After 5 min, the absorbance was read at 510 nm using a Jasco V160 UV-Vis spectrophotometer. TFC was determined using the calibration curve obtained with catechin and described as milligrams of catechin equivalents (CE) per gram of *Ephedra alata*.

### 2.4 HPLC analysis

HPLC was carried out with a Shimadzu Prominence modular HPLC system equipped with an online degasser, a thermostatic column compartment, and a SPD-20A UV detector for flavonoid and phenolic acid separation and analysis at 268 nm. A Shim-pack VP-ODS C18 analytical column (5 μm, 4.6–250 mm) from Shimadzu Co., Japan was used. The gradient system used 0.2% acetic acid in water (B) and acetonitrile (A). Before use, the mobile phase components were sonicated, filtered through 0.45 μm membranes, and then delivered from the solvent reservoir to the column at a flow rate of 1 mL. min^-1^. The elution involved a linear gradient (see Table 1). The injection volume was 10 μL and the column was at room temperature. Before injection, the column was equilibrated and the mobile phase lasted 40–50 min. Polyphenol solutions were freshly prepared before use by diluting the polyphenol solution with the mobile phase according to established protocols (Bouaziz et al., 2015; Saleem et al., 2020). The collected data included peak area and retention periods, with three 20-μL injections of each solution. The sample (10 mg of plant extract) was dissolved in 10 mL of HPLC-grade methanol and filtered through a 0.45 μm Millipore nylon filter disc; 20 μL of the filtered sample was used for the HPLC analysis. The mobile phase (0.2% of acetic acid and acetonitrile) was sonicated and filtered through a Whatman RC55 membrane before injection in the column at a flow rate of 1 mL min^-1^. The effluent was detected at a wavelength of 268 nm (Zeghoud et al., 2021).

**TABLE 1:** Gradient for phenolic acid elution.

Time (min)	CH_3_COOH v/v%	CH_3_CN v/v%
0	90	10
6	86	14
16	83	17
23	81	19
28	77	23
35	60	40
38	90	10
50	90	10

### 2.5 DPPH antioxidant assay

The DPPH assay was used to determine *Ephedra alata* extract antioxidant potential following the procedure outlined by Thaipong et al. (Thaipong et al., 2006). Briefly, 150 μL of leaf extract was incubated with a DPPH solution (25 mg DPPH in 100 mL methanol and diluted to obtain an absorbance of 1.1 ± 0.02 at 515 nm) for 30 min, and then absorbance was quantified at 515 nm using UV- Visible (UV-Vis) spectrometry (Figure 2). Ascorbic acid was used as standard reference, with values ranging from 0.003 to 0.12 mg.mL^-1^ in our experimental settings. DPPH inhibition percentage by the extract was determined with Eq 1:

$$\text{Inhibition}=\left[\frac{A_{\text{control}}\;–\;A_{\text{sample}}}{A_{\text{control}}}\right]\times 100$$ (1)

The sample concentration that inhibited 50% of DPPH (IC_50_) was determined by analysis of the dose-response curve (Ziani et al., 2019).

### 2.6 Total antioxidant capacity

The total antioxidant capacity was valuated with the phosphomolybdenum method in which Mo (VI) is converted to Mo (V) through a reduction reaction facilitated by the plant extract. Consequently, a green phosphate/Mo(V) complex is produced at low pH. Each sample aliquot (0.1 mL) was combined with 1 mL of reagent solution (0.6 M H_2_SO_4_, 28 mM Na_3_PO_4_, and 4 M Al_2_(MoO_4_)_3_) and incubated at 95°C for 90 min. After cooling the samples to room temperature, absorbance was measured at 695 nm. The blank solution contained 1 mL of methanol alone. The total antioxidant capacity was expressed in mg (gallic acid equivalent) GAE. g^-1^ dry weight (Ziani et al., 2019). Ascorbic acid was employed as reference standard due to its strong reducing properties, widespread availability, and ease of obtention, making it a suitable choice for standardization in experimental setups.

### 2.7 Antibacterial activity

The extract antibacterial activity against *Staphylococcus aureus* (ATCC 25923) and *Pseudomonas aeruginosa* (ATCC 27853) was assessed using the agar well diffusion method. Bacterial strains were cultured on agar plates by adding 100 μL of the bacterial strain broth and allowing them to grow for 24 h. After this initial growth period, wells of 6 mm in diameter were created in the agar using a sterile stainless steel cork borer. Then, various concentrations of the extract (1, 0.5, 0.25, and 0.125 mg.mL^-1^ in DMSO) were added to the wells (5 µL of extract/well). The experimental setup included a comparison with ciprofloxacin, a known antibacterial agent (Figure 3 and Table 2). The plates were then incubated at 37°C for 24 h to allow bacterial growth and interaction with the plant extract.

**TABLE 2:** Antibacterial effect of the *Ephedra alata* leaf extract. The inhibition zone represents the mean value of three independent experiments.

Sample	Conc	Zone of inhibition (mm)	
		Gram-negative	Gram-positive
		*Pseudomonas aeruginosa*	*Staphylococcus aureus*
*Ephedra alata* leaf extract^a^	1 mg.mL^-1^	19.0±0.2	13.0±0.1
	0.5 mg.mL^-1^	16.0±0.1	12 .0±0.1
	0.25 mg.mL^-1^	13 .0±0.1	10 .0±0.1
	0.125 mg.mL^-1^	11.0±0.2	9 .0±0.2
Ciprofloxacin	50 µg	22.0± 0.4	14.0± 0.2

^a^DMSO, showed no antibacterial activity and had no effect on the zone of inhibition.

### 2.8 ZnO NP synthesis and characterization

**FIGURE 1:**
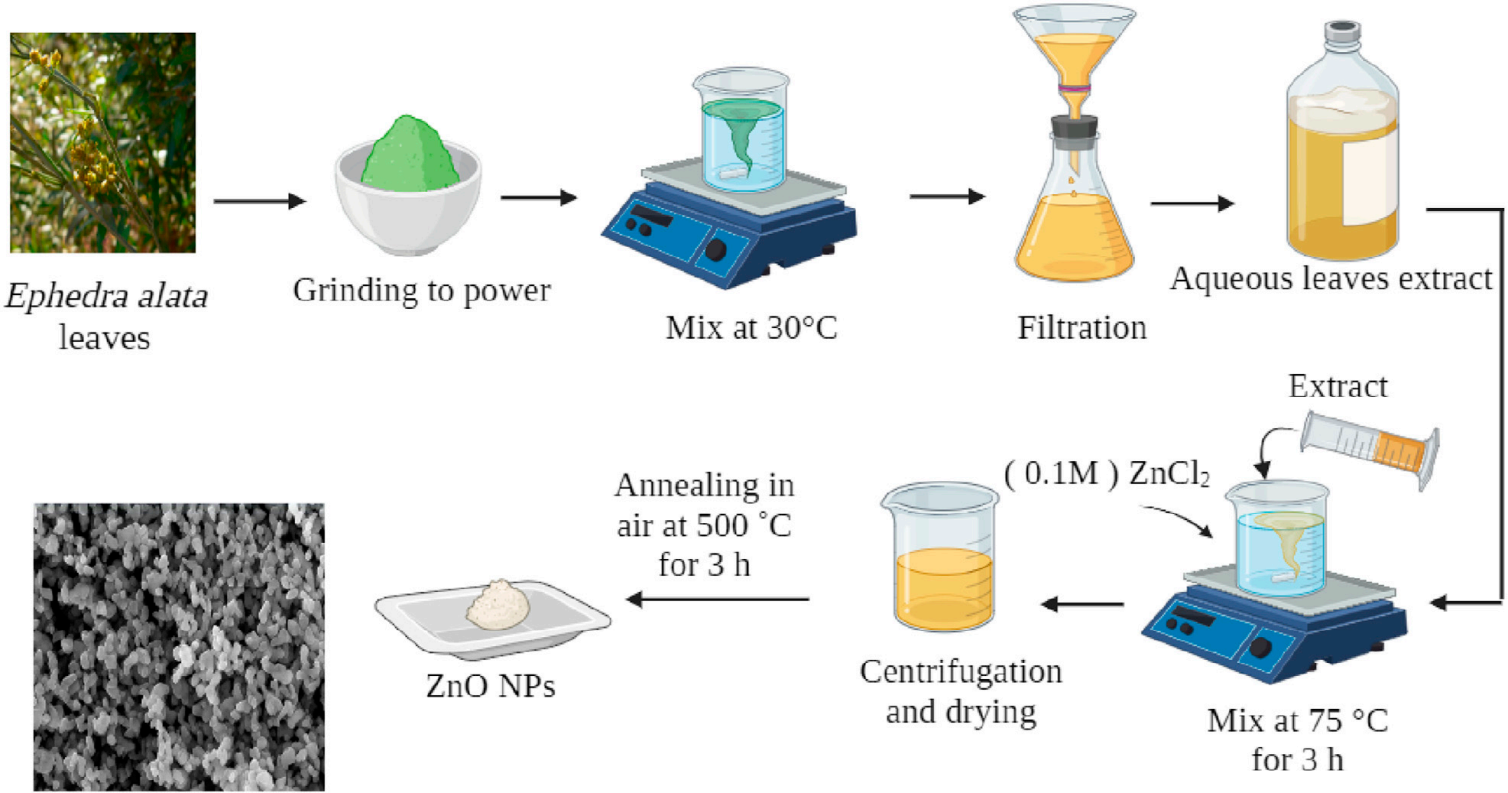
ZnO NP synthesis using *Ephedra alata* leaf extract.

A previously described technique, with minor modifications, was used for ZnO NP synthesis. A 0.1M ZnCl_2_ solution was added to 100 mL of leaf extract (Figure 1). The resulting mixture was continuously stirred at 75°C for 3 h. After the reaction completion, the mixture was cooled to 25°C and then centrifuged (10,000 rpm for 10 min) to separate the liquid component. The solid substance was washed with distilled water three times. The washed material was dried in a sterile Petri dish and then oven dried at 90°C, followed by grinding using a mortar and pestle and calcination at 500°C for 3 h to eliminate any extraneous element or impurity (Jan et al., 2020).

The ZnO NP crystalline structure was investigated by X-ray diffraction (XRD) using a Rigaku Miniflex 600 apparatus. The UV-Vis spectra were obtained with a Jasco V160 UV-Vis spectrophotometer using 0.1 mg of ZnO NPs in 2 mL distilled water. The Tauc relationship [(αhν) = A (hν - Eg)^n^] was used to calculate the bandgap energy (Eg), where α is the absorption coefficient, h is the Planck constant, A indicates a constant, and n is a constant with a value of 2 (Ilham et al., 2023). A Nicolet iS50 Fourier-transform infrared (FTIR) spectrometer and the potassium bromide method were used to obtain the IR spectra (Barhoum and García-Betancourt, 2018). Scanning electron microscopy (SEM) (FESEM, Leo Supra 55-Zeiss Inc., Germany) allowed determining the NP elemental composition, morphology, and size.

### 2.9 Photocatalytic activity

To assess ZnO NP photocatalytic activity, the degradation of MB in an aqueous solution was monitored after exposure to a 1000 W UV lamp (Senthilraja et al., 2014). To optimize MB dye degradation, a MB dye solution was prepared with a concentration of 6 × 10^5^ M, and 30 mg of ZnO NPs was added to the solution. The solution was left in the dark for 15 min before UV exposure. The reaction progress was monitored by UV-Vis spectrometry of aliquots taken at specific intervals (0, 15, 25, 35, 45, 60, and 90 min). The reduction process was carried out entirely under UV light, and the blue color intensity in the reaction mixture gradually decreased. Each aliquot was centrifuged to stop the reaction and the absorbance was measured. The λ_max_ was 663 nm. The degradation efficacy (%) was determined with the following equation (Ben Amor et al., 2022):

$$\text{Degradation ratio }\left(\%\right)=\frac{\left(C_{0}-C_{t}\right)}{C_{0}}\times 100$$ (2)

where, C_0_ is MB concentration at time 0, and C_t_ the concentration at a given time point.

## 3 Results and discussion

### 3.1 Qualitative analysis of the *Ephedra alata* leaf extract

The qualitative tests carried out using the *Ephedra alata* leaf extract showed the presence of tannins, alkaloids, terpenoids, flavonoids, coumarins, glycosides, saponins, and volatile oils (Table 3). According to these qualitative tests, flavonoids and glycosides were the most abundant components. These findings are in line with the results reported by Naim et al. (Kittana et al., 2017) who identified different organic compounds in E*phedra alata* aqueous extracts. As many phytochemicals with therapeutic properties are abundantly present in *Ephedra alata,* it is important to explore their use in medicine.

**TABLE 3:** Results of the saponin, alkaloid, glycoside and coumarin tests.

Phytochemical	Observation	Concentration
Tannins	Bluish-green color	++
Alkaloids	White precipitate	++
Terpenoids	Reddish-brown	++
Flavonoids	Intense red	+++
Coumarins	Red	++
Glycosides	Brown	+++
Saponins	Forms foam in all tubes	++
Volatile oils	Golden/light brown color	++

(+): trace amount; (++): moderately present; (+++): highly present.

The estimated TPC of the leaf extract was 48.7 ± 0.9 mg GAE. g^-1^. For comparison, *Ephedra alata* Decne from Jordan displayed TPC values of 16.2 and 11.9 mg GAE. g^-1^ when using methanolic and aqueous extracts, respectively (Al-Rimawi et al., 2017). *Ephedra pachyclada* from Iran had a TPC of 45 mg GAE. g^-1^ dry weight (Ghasemi et al., 2013). Notably, this study highlighted the abundance of phenolic compounds in the plant (48.7 mg.g^-1^), surpassing the quantities found in guava (1.3–2.5 mg.g^-1^) and plum fruits (1.3–3.7 mg.g^-1^) (Thaipong et al., 2006). Moreover, the estimated TFC was 1.7 ± 0.4 mg g^-1^. As the concentration of these compounds is strongly influenced by the environmental conditions (Al-Rimawi et al., 2017), the optimal harvesting time to extract phenolic and flavonoid compounds is during the warmer season. A study on *Ephedra vulgaris* from India reported a TFC of 1.5 ± 0.2 mg.g^-1^. These data indicate similar flavonoid levels in ephedra from different geographical regions.

Table 4 presents the phenolic compound content in the *Ephedra alata* leaf extract determined by HPLC. This analysis showed also the absence of caffeic acid, naringin, and quercetin in the sample. The concentrations listed in Table 4 were determined using a standardized methodology and a standard curve. The total phenolic content was 1.5 μg.g^-1^.

**TABLE 4:** Phenolic compounds identified by HPLC in the *Ephedra alata* leaf extract.

Compounds	Content (g)
Gallic acid	304.4 µg^-1^
Chlorogenic acid	815.1 µg^-1^
Vanillic acid	215.8 µg^-1^
Caffeic acid	0.0 µg^-1^
Vanillin	33.4 µg^-1^
p-Coumaric acid	15.4 µg^-1^
Rutin	93.5 µg^-^
Naringin	0.0 µg^-1^
Quercetin	0.0 µg^-1^
Total	1.5 mg^-1^

### 3.2 Antioxidant activity of the *Ephedra alata* leaf extract

**FIGURE 2:**
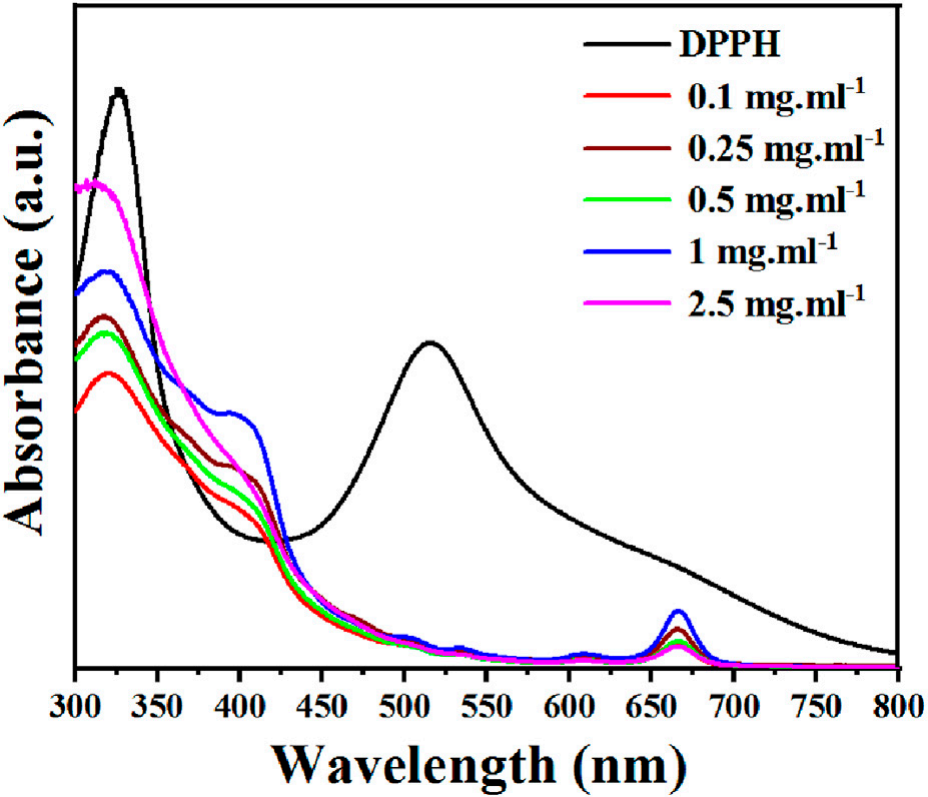
DPPH inhibition rate by the *Ephedra alata* leaf extract sample at the indicated concentrations.

Phenolic compounds have redox properties and act as effective neutralizers of oxygen radicals by serving as singlet oxygen quenchers, hydrogen donors, and reducing agents. For example, flavonoids and polyphenols act as radical scavengers and inhibit oxidative stress by donating electrons to stabilize and neutralize free radicals. The essential oils, and other phytochemicals, contribute to the overall antioxidant defense mechanism (Ghasemi et al., 2013). *Ephedra alata* antioxidant activity is essential for the plant defense against environmental challenges, but also holds potential therapeutic implications for human health because oxidative stress is implicated in various diseases. Therefore, the antioxidant potential of the *Ephedra alata* leaf extract was tested with the DPPH scavenging assay, a widely employed technique to quantify free radical neutralization (Baliyan et al., 2022). DPPH interaction with the extract led to a color change from purple to yellow as protons were donated, resulting in a decrease of DPPH absorption. The extract demonstrated a concentration-dependent DPPH scavenging activity inhibition (y = 0.5944x + 2.216, R = 0.99) (Figure 2). At the highest concentration (2.5 mg.mL^-1^), the extract inhibited 70.5% of DPPH activity, as reported by previous studies (Jaradat et al., 2021; Atri et al., 2023). This indicates high amounts of antioxidants, flavonoids, and phenols in *Ephedra alata* from different regions.

The total antioxidant activity of the *Ephedra alata* leaf extract reflects its capacity to counteract harmful free radicals implicated in cell damage and various health disorders. The molybdenum assay was used to assess the leaf extract antioxidant activity, specifically the ability to transfer electrons and hydrogen to neutralize free radical damage (Chen et al., 2021). The total antioxidant activity of the *Ephedra alata* leaf extract sample was 49.5 ± 3.4 mg.g^-1^, consistent with previous research (Al-Rimawi et al., 2017). The findings of the present study suggest that the high flavonoid content in the extract (Table 3) contributes to its high antioxidant capacity (Benabderrahim et al., 2019; Soumaya et al., 2020; Al-Nemi et al., 2022).

### 3.3 *Ephedra alata* leaf extract antibacterial activity

**FIGURE 3:**
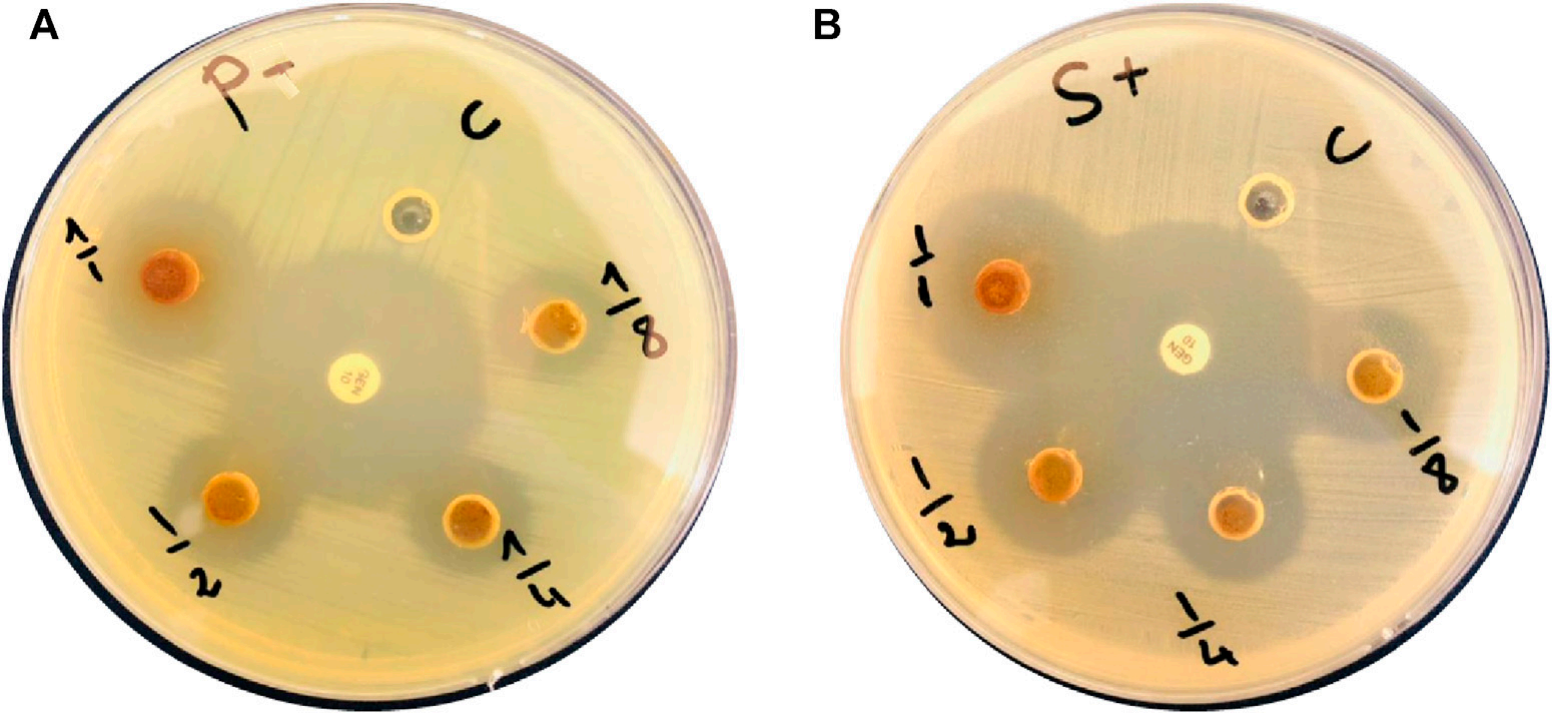
*Ephedra alata* leaf extract bactericidal effect against **(A)** *Pseudomonas aeruginosa* and **(B)** *Staphylococcus aureus*.

Assessment of the extract antibacterial activity against Gram (+) and (−) bacteria revealed a concentration-dependent increase of the inhibition zones (Figure 3, Table 2). For the Gram (+) *S. aureus*, higher extract concentrations led to larger inhibition zones, from 9 ± 0.2 mm at 0.125 mg.mL^-1^ to 13 ± 0.1 mm at 1 mg.mL^-1^. Similarly, for the Gram (−) *P. aeruginosa*, the inhibition zone increased with higher extract concentrations, reaching 19 ± 0.2 mm at 1 mg.mL^-1^. In a previous study, a methanol extract of *Ephedra alata* gave inhibition zones ranging from 0 to 17 mm for *S. aureus* and *Staphylococcus epidermidis* (Gram (+)) and for *Escherichia coli* and *P. aeruginosa* (Gram (−)) (Salman et al., 2021). Antimicrobial properties of water, methanol, and acetonitrile extracts of *Ephedra alata* against were previously examined four bacteria (*S. aureus*, *Bacillus subtilis*, *P. aeruginosa*, *E. coli*) and four fungi (*Penicillium italicum*, *Syncephalastrum racemosum*, *Aspergillus fumigatus*, *Candida albicans*) (Danciu et al., 2019). They authors found that only the acetonitrile extract displayed activity against both bacteria and fungi. The methanolic extract showed only antifungal activity against *A. fumigatus* and *P. italicum* (Danciu et al., 2019). Our *Ephedra alata* leaf extract exhibited antimicrobial properties owing to its rich phytochemical composition, including alkaloids (ephedrine and pseudoephedrine), flavonoids, tannins, polyphenols, and essential oils. Alkaloids disrupt the microbial cell membrane and metabolic processes, while flavonoids and polyphenols act as antioxidants with antimicrobial effects. Tannins precipitate proteins, leading to microbial cell damage. Essential oils contribute volatile compounds with antibacterial and antifungal activities. The synergistic effects of these bioactive compounds enhance the overall antimicrobial potency of the extract. *Ephedra alata* traditional medicinal use for treating infectious diseases further underscores its antimicrobial potential (Altemimi et al., 2017).

### 3.4 Characteristics of the synthesized ZnO NPs

**FIGURE 4:**
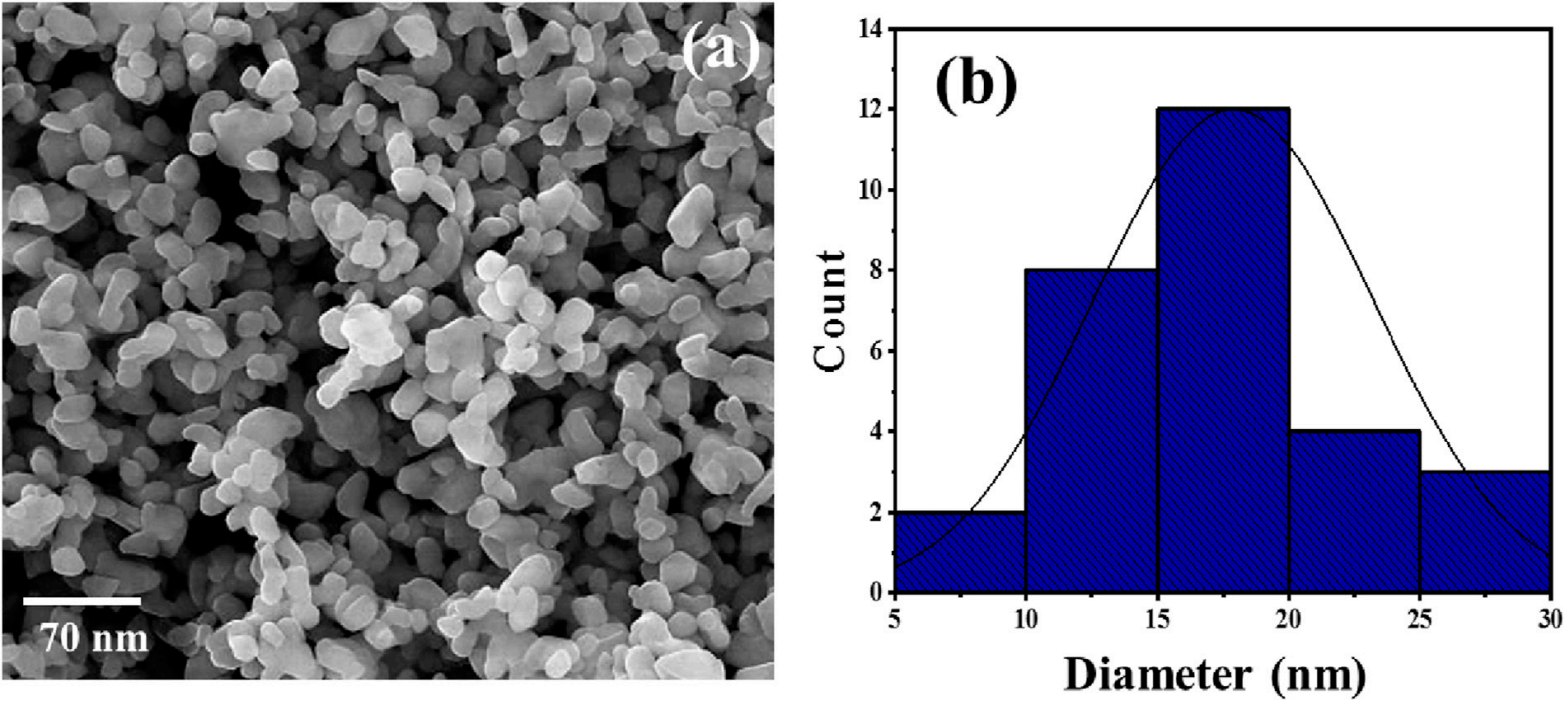
Representative SEM photograph **(A)** and size distribution **(B)** of ZnO NPs synthesized in the presence of the *Ephedra alata* leaf extract.

ZnO NP production typically began by mixing the *Ephedra alata* leaf extract with a ZnCl_2_ solution. The different phytochemicals in the extract (e.g., alkaloids, flavonoids, and phenolic compounds) worked as reducers and stabilizers. These compounds play a crucial role in zinc ion reduction, thereby initiating ZnO NP formation. The reduction process, often carried out at a specific temperature and pH, leads to nucleation and growth of ZnO NPs. The phytochemicals in the plant extract contribute to control the NP size, shape, and crystallinity. The specific mechanism may involve the interaction of functional groups in the plant extract with zinc ions, resulting in the production of stable ZnO NPs through a green and sustainable approach. The SEM analysis revealed that all ZnO NPs had a spherical shape, with an average diameter of 20 nm (Figure 4).

**FIGURE 5:**
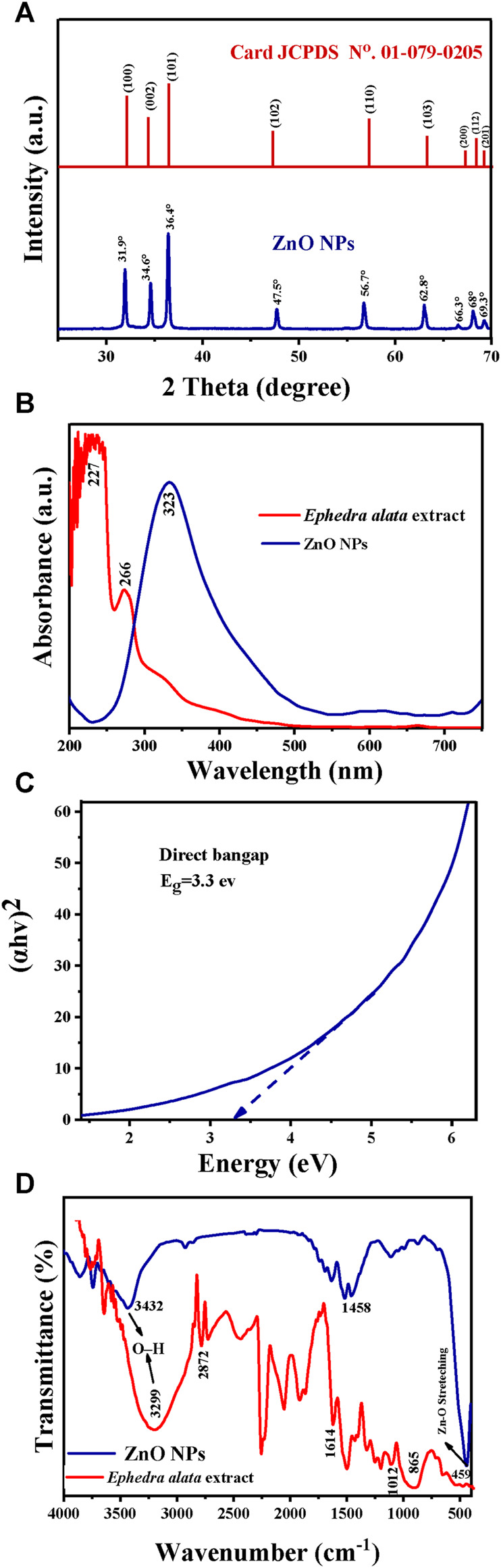
Characteristics of the *Ephedra alata* leaf extract and plant-based ZnO NPs: **(A)** XRD patterns of the ZnO NPs; **(B)** UV-Vis spectra of the *Ephedra alata* leaf extract and ZnO NPs; **(C)** Optical energy bandgap of ZnO NPs; **(D)** FTIR spectra of the *Ephedra alata* leaf extract and ZnO NPs.

The XRD pattern of these ZnO NPs included distinct peaks at 2θ: 31.9°, 34.6°, 36.4°, 47.5°, 56.7°, 62.8°, 66.3°, 68°, and 69.3° that matched the crystallographic planes (100), (002), (101), (102), (110), (103), (200), (112), and (201), respectively (Figure 5A). Comparison of these results with the JCPDS data sheet/ICDD no. 01–079–0205 confirmed the presence of a wurtzite hexagonal structure in the ZnO NPs. Moreover, the sharp, well-defined, and intense diffraction reflections confirmed the NP crystalline nature. Their mean crystallite size (D; ∼25 nm) was calculated with the Debye–Scherrer’s equation (D = Kλ/(β-cosθ)), where D is the average crystallite size, λ is the X-ray wavelength (0.15406 nm), K is a constant (0.9), β is the full width at half maximum of the peak centered at 20°, and θ is the Bragg angle.

The UV-Vis spectra of the *Ephedra alata* leaf extract included prominent peaks at 227 nm (indicative of the presence of condensed tannins) and 266 nm (presence of polyphenols) (Figure 5B). These compounds have antioxidant activity and play a stabilizing role, preventing NP agglomeration. The UV-Vis spectra of the obtained ZnO NPs had a distinct peak at 323 nm, in agreement with the findings by Vaseem et al. (Vaseem et al., 2012; Vijayakumar et al., 2018). ZnO NPs are effective photocatalysts due to their significant bandgap, particularly in the presence of UV radiation. Their efficacy is influenced by the electronic band structure, bandgap energy, and UV-Vis absorption properties. Previous studies showed that the photonic bandgaps of ZnO nanostructures range between 2.7 eV and 4.7 eV. The bandgap energy of the produced ZnO NPs was 3.3 eV and was determined from the curve of (hv)^2^ in function of the energy (eV) (Figure 5C).

The FITR spectra (Figure 5D; Table 5) included the characteristic peaks observed in the *Ephedra alata* leaf extract at 3,299, 2,872, 1,614, 1,012, and 865 cm^−1^. The peak at 3,299 cm^−1^ is due to the hydroxyl (OH) group stretching vibration resulting from inter- and intra-molecular hydrogen bonding (Atri et al., 2023; Abdelbaki et al., 2024). The peak at 2,872 cm^−1^ indicates the stretching of CH groups in free sugars (Seghir et al., 2023). The peak at 1,614 cm^−1^ is due to carbonyl ester (C=O) functional groups (Seghir et al., 2023). The large peak between 1,617 cm^−1^ and 1700 cm^−1^ indicates the existence of bound water, and the area from 865 to 1,010 cm^−1^ displayed carbohydrate fingerprints, indicating the presence of the functional groups of polysaccharides involving various molecular vibrations, such as stretching (C-O-C), bending (O-H), and deforming (CH_3_) vibrations. Peaks below 1,012 cm^−1^ indicated potential connections between monosaccharide molecules (Faisal et al., 2021). For ZnO NPs, the broad peak at 3,432 cm^−1^ and the peak at 1,458 cm^−1^ are due to OH group stretching and amine (–NH) vibrational stretching in protein amide bonds, respectively (Rabecca et al., 2022; Amor et al., 2023). The peaks at 1,110 cm^−1^ and 1,453 cm^−1^ denote the presence of alcohol and phenolic compounds, and C-N bond stretching vibrations in aromatic amines, respectively (Kalaimurugan et al., 2022). The peak at 459 cm^−1^ is due to Zn-O bond stretching vibrations, indicative of ZnO bonds (Thi et al., 2020; Rahman et al., 2022; Ishwarya et al., 2023).

**TABLE 5:** FTIR peak assignment for the *Ephedra alata* leaf extract and ZnO NPs.

Frequency (cm)	Assignment	Ref.
3299^–1^	OH stretching vibrations	Atri et al. (2023)
2872^–1^	C-H groups	Seghir et al. (2023)
1614^–1^	Carbonyl ester (C=O) functional groups	(Seghir et al., 2023)
3432^–1^	OH group stretching	Amor et al. (2023)
1458^–1^	Amine (–NH) vibrational stretching	Rabecca et al. (2022)
1110^–1^	Presence of alcohol and phenolic compounds	Kalaimurugan et al. (2022)
1453^–1^	C-N bond stretching vibrations in aromatic amines	Dappula et al. (2023)
459^–1^	Zn-O bond stretching vibrations	Dappula et al. (2023)

### 3.5 Photocatalytic degradation of MB

**FIGURE 6:**
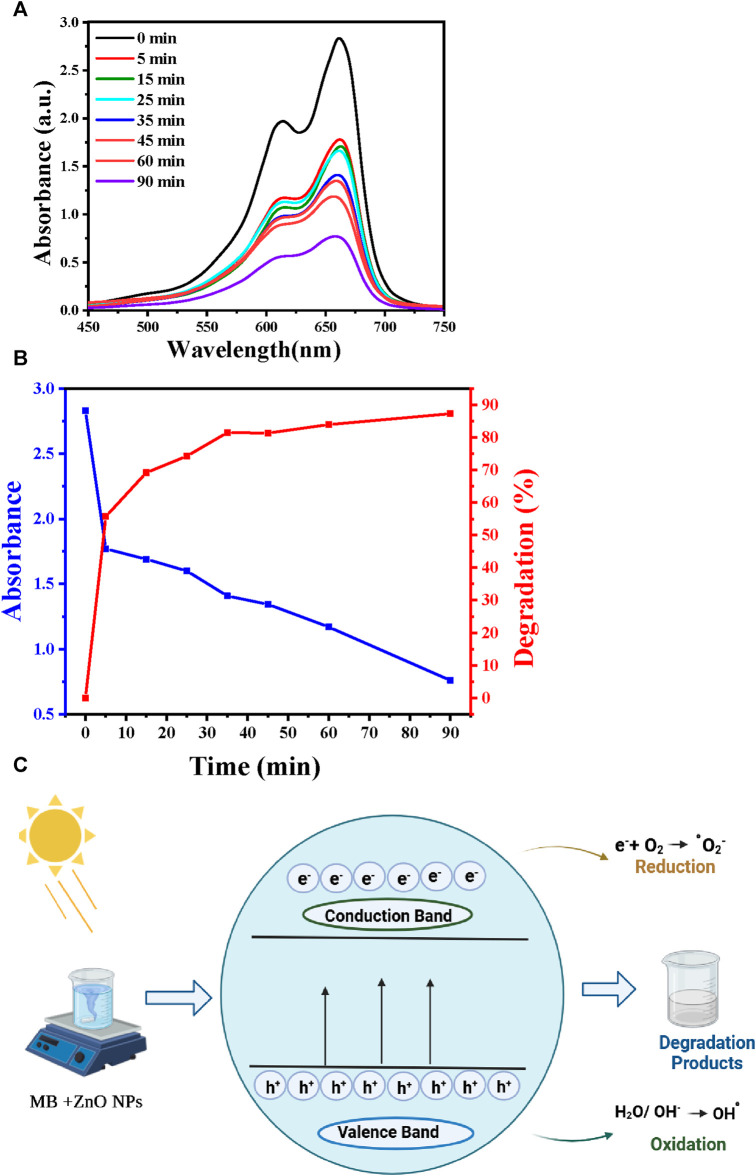
MB photocatalytic removal. **(A)** UV-Vis spectra showing MB degradation in function of the reaction time. **(B)** Correlation between degradation efficiency, UV-Vis absorption, and reaction time. **(C)** Schematic representation of MB photodegradation in the presence of ZnO NPs.

MB was used as organic pollutant to evaluate ZnO NP photocatalytic activity when exposed to UV light. MB is a poisonous, carcinogenic, and non-biodegradable compound (Mageshwari et al., 2013). Thus, MB removal from wastewater requires the development of efficient and environmentally friendly techniques, for instance photocatalytic degradation. This technique decreases processing costs and leads to the complete mineralization of MB into non-toxic species. In optimal experimental conditions (90 min contact time, pH 7, and 25°C), 87% of MB was degraded in the presence of ZnO NPs (Figures 6A, B). Table 6 presents a comparison of the results obtained on the effectiveness of MB dye removal using ZnO NPs synthesized using different extracts. The ZnO NPs synthesized in this study showed a very high removal efficiency in a relatively short period (90 min).

**TABLE 6:** Comparison of the effectiveness of different ZnO NPs for azo dye removal.

NP source	Zn salt	ZnO NP size (nm)	Dye degradation conditions						Ref.
			Azo dye	Concentration	Volume	Catalyst mass (mg)	Time (min)	Dye removal (%)	
*Calligonum comosum L*	ZnCl_2_	32.8	Methylene blue	2.50 × 10^−5^ M	30 mL	50	120	86	Gharbi et al. (2023)
			Rose bengal					92	
*Syzygium cumini*	Zn(NO_3_)_2_·2H_2_O	12.5	Methylene blue	40 ppm	500 mL	100	180	91	Sadiq et al. (2021)
Shrimp shell chitosan	ZnCl_2_	30.9	Methylene blue	6 × 10^−5^ M	30 mL	30	60	60	Ben Amor et al. (2022)
*Cocos nucifera*	Zn(NO_3_)_2_·2H_2_O	16.6	Methylene blue	50 mg.L^−1^	20 mL	5	60	84	Rahman et al. (2022)
*Myristica fragrans*	Zn(NO_3_)_2_·2H_2_O	41.2	Methylene blue	20 ppm	45 mL	25	140	80	Faisal et al. (2021)
*Pluchea indica*	(Zn (CH_3_COO)_2_.2H_2_O)	21.9	Methylene blue	10 mg.L^−1^	50 mL	100	150	95.3	Al-Askar et al. (2023)
*Ephedra alata*	ZnCl_2_	25 nm	Methylene blue	6 × 10^5^ M	40	30	90	87	This work

Figure 6C illustrates the mechanism by which azo dyes undergo photocatalytic degradation in the presence of the ZnO photocatalyst and of UV irradiation. Typically, electrons move from the valence to the conduction band when the photocatalyst is exposed to UV radiation. The associated energy exceeds the ZnO bandgap (3.3 eV). This promotes electron (e−) generation in the conduction band and hole (h^+^) creation in the valence band (Osuntokun et al., 2019). The formed holes might directly oxidize the adsorbed dye or react with hydroxyl (OH-) or H_2_O, resulting in the generation of hydroxyl radicals (·OH). The generated electrons facilitate the reduction of molecular oxygen (O_2_) that is adsorbed on the photocatalyst surface, resulting in superoxide radical (·O_2_^−^) production. The generated hydroxyl (·OH) and superoxide radicals (·O_2_−) lead to the dye degradation. The reaction Eqs 4–8 are presented below (Rathnasamy et al., 2017).

$$ZnO+h\upsilon \to ZnO\;\left(h_{VB}^{+}+e_{CB}^{-}\right)$$ (4)

$$h_{VB}^{+}+H_{2}O\to H^{+}+•OH$$ (5)

$$e_{CB}^{-}+O_{2}\to {O_{2}}^{-}$$ (6)

$${OH}^{-}\to h_{VB}^{+}+•OH$$ (7)

$$•OH+B\;dey+O_{2}\;\to Organic\;ions+H_{2}O+CO_{2}{+H}_{2}O$$ (8)

Numerous studies have highlighted the efficacy of ZnO NPs as photocatalysts for organic dye degradation, notably MB. However, their practical application necessitates recurrent use, prompting researchers to explore methods for assessing and enhancing ZnO NP recyclability. Agglomeration, surface fouling, and crystal structure changes have been identified as contributors to the ZnO NP photocatalytic efficiency decline over successive cycles. Different strategies (e.g., washing and calcination) have been investigated for ZnO NP regeneration, offering insights into sustainable and cost-effective photocatalytic processes for water purification (Mahlaule-Glory and Hintsho-Mbita, 2022). Saied et al. (Saied et al., 2022) emphasized the importance of light stimulators for the efficient degradation of the crystal violet dye using biosynthesized hematite NPs. Moreover, optimal dye decolorization was observed with higher ZnO NP concentrations, and this was attributed to the higher number of adsorption sites (Rasool et al., 2023). The degradation time remained constant for pure dyes compared with complex solutions, but efficiency declined with higher dye concentrations, suggesting competition for limited adsorption sites (Rasool et al., 2023). Another study also showed that the active sites on the ZnO NP surfaces decrease with higher adsorbent quantity, reducing their capacity to adsorb dye molecules (Al-Askar et al., 2023).

Omran et al. (Omran, 2023) reported superior eco-bioremediation features of ZnO NPs and high capacity to degrade MB (88.93%) and crystal violet (80.69%). Nguyen et al. (Nguyen et al., 2023) demonstrated that ZnFe_2_O_4_@ZnO nanocomposites (5.0 mg.L^−1^ with 0.33 g.L^−1^ of ZnO) degraded 94.85% of a Congo red dye solution. Gatou et al. (Gatou et al., 2023) described the reusability of optimized ZnO NP samples under UV light for five consecutive photocatalytic cycles, maintaining exceptional resistance to degradation. Khan et al. (Khan et al., 2021) evaluated ZnO NPs synthesized using *Passiflora foetida* peels and found that decomposition efficiency for MB and rhodamine-B dyes decreased over five cycles. Abdulaziz et al. (Al-Askar et al., 2023) found that ZnO NPs manufactured in the presence of *Pluchea indica* leaves removed 87% of dye after four reuses. Another study (Alzahrani et al., 2023) tested the ZnO-Ag composite recyclability as nanophotocatalyst for MB dye degradation and showed degradation of 75%–98% over four cycles, indicating its potential for repeated use in photodegradation processes.

Table 6 provides a comparative assessment of the effectiveness of azo dye removal, considering ZnO nanoparticles synthesized using various extracts. The extracts utilized include Calligonum comosum L., Syzygium cumini, shrimp shell chitosan, Cocos nucifera, Myristica fragrans, and Ephedra alata (Faisal et al., 2021; Sadiq et al., 2021; Rahman et al., 2022; Gharbi et al., 2023). Key parameters considered in the analysis encompass NP source, synthesis method (e.g., ZnCl_2_ for Ephedra alata), ZnO NP size, azo dye type and its initial concentration, solution volume, catalyst mass, reaction time, and the percentage of dye removal. This comparison highlighted the azo dye (MB) removal efficiency (87% in 90 min) of the ZnO NPs produced in the presence of *Ephedra alata* leaf extract. This performance was similar or higher than that of ZnO NPs synthesized in the presence of other sources. This indicates the potential of *Ephedra alata* as a source for ZnO NP synthesis with promising applications in azo dye degradation, contributing valuable insights into the broader field of NP-based environmental remediation.

## 4 Conclusion

In this study, phytochemicals were successfully extracted from *Ephedra alata* leaves and thoroughly analyzed using HPLC. The results showed that these leaves contained key phenolic acids: chlorogenic acid, gallic acid, vanillic acid, vanillin, p-coumaric acid, and rutin. The TPC of the plant extract was 48.7 ± 0.9 mg.g^-1^ and the TFC was 1.7 ± 0.4 mg.g^-1^. The leaf extract exhibited remarkable antioxidant activity, as indicated by the DPPH inhibition rate of 70.5% and the total antioxidant activity of 49.5 ± 3.4 mg.g^-1^. The *Ephedra alata* leaf extract also displayed good antibacterial activity against Gram (+) and particularly Gram (−) bacteria. Moreover, this extract was used for the environmentally friendly production of ZnO NPs, avoiding the use of toxic chemicals. The obtained ZnO NPs had a spherical morphology with particle size ranging from 5 to 30 nm and crystallite size of 25 nm, a well-defined wurtzite crystal structure, and a bandgap energy of 3.3 eV. In specific conditions (90 min contact time, pH 7, and 25°C), the ZnO NPs degraded 87% for MB in aqueous solution, indicating their potential application in sustainable water treatment and pollution control methods.

## Data Availability

The original contributions presented in the study are included in the article/Supplementary material, further inquiries can be directed to the corresponding author.

## References

[B1] Abdelbaki H. Djemoui A. Souli L. Souadia A. Ouahrani M. R. Djemoui B. (2024). Plant mediated synthesis of flower-like Cu2O microbeads from artimisia campestris L. Extract for the catalyzed synthesis of 1, 4-disubstituted 1, 2, 3-triazole derivatives. Front. Chem. 11, 1342988. 10.3389/fchem.2023.1342988 38298761 PMC10829102

[B2] Ahmed Z. Aziz S. Hanif M. Mohiuddin S. G. Khan S. H. A. Ahmed R. (2020). Phytochemical screening and enzymatic and antioxidant activities of Erythrina suberosa (Roxb) bark. J. Pharm. bioallied Sci. 12, 192. 10.4103/jpbs.jpbs_222_19 32742119 PMC7373117

[B3] Ajayan A. S. Hebsur N. (2020). Green synthesis of zinc oxide nanoparticles using neem (Azadirachta Indica) and Tulasi (Ocimum tenuiflorum) leaf extract and their characterisation. Int. J. Curr. Microbiol. Appl. Sci. 9, 277–315. 10.20546/ijcmas.2020.902.035

[B4] Alallam B. Doolaanea A. A. Alfatama M. Lim V. (2023). Phytofabrication and characterisation of zinc oxide nanoparticles using pure curcumin. Pharmaceuticals 16, 269. 10.3390/ph16020269 37259414 PMC9960272

[B5] Al-Askar A. A. Hashem A. H. Elhussieny N. I. Saied E. (2023). Green biosynthesis of zinc oxide nanoparticles using Pluchea indica leaf extract: antimicrobial and photocatalytic activities. Molecules 28, 4679. 10.3390/molecules28124679 37375234 PMC10304739

[B7] Al-Mur B. A. (2023). Green zinc oxide (ZnO) nanoparticle synthesis using mangrove leaf extract from avicenna marina: properties and application for the removal of toxic metal ions (Cd2+ and Pb2+). Water 15, 455. 10.3390/w15030455

[B8] Al-Nemi R. Makki A. A. Sawalha K. Hajjar D. Jaremko M. (2022). Untargeted metabolomic profiling and antioxidant capacities of different solvent crude extracts of Ephedra foeminea. Metabolites 12, 451. 10.3390/metabo12050451 35629955 PMC9146585

[B9] Al-Radadi N. S. (2023). Ephedra mediated green synthesis of gold nanoparticles (AuNPs) and evaluation of its antioxidant, antipyretic, anti-asthmatic, and antimicrobial properties. Arabian J. Chem. 16, 104353. 10.1016/j.arabjc.2022.104353

[B10] Al-Rimawi F. Abu-Lafi S. Abbadi J. Alamarneh A. A. Sawahreh R. A. Odeh I. (2017). Analysis of phenolic and flavonoids of wild Ephedra alata plant extracts by LC/PDA and LC/MS and their antioxidant activity. Afr. J. Traditional, Complementary Altern. Med. 14, 130–141. 10.21010/ajtcam.v14i2.14 PMC5446436 28573229

[B11] Altemimi A. B. Lakhssassi N. Baharlouei A. Watson D. Lightfoot D. (2017). Phytochemicals: extraction, isolation, and identification of bioactive compounds from plant extracts. Plants 6 (4), 42. 10.3390/plants6040042 28937585 PMC5750618

[B12] Alzahrani E. A. Nabi A. Kamli M. R. Albukhari S. M. Althabaiti S. A. Al-Harbi S. A. (2023). Facile green synthesis of ZnO NPs and plasmonic Ag-supported ZnO nanocomposite for photocatalytic degradation of methylene blue. Water 15, 384. 10.3390/w15030384

[B13] Amor I. B. Hemmami H. Laouini S. E. Ahmed S. Mohammed H. A. Abdullah J. A. A. (2023). Enhancing oxidant and dye scavenging through MgO-based chitosan nanoparticles for potential antioxidant coatings and efficient photocatalysts. Biomass Convers. Biorefinery 2023, 1–15. 10.1007/s13399-023-04923-1

[B14] Antonio-Pérez A. Durán-Armenta L. F. Pérez-Loredo M. G. Torres-Huerta A. L. (2023). Biosynthesis of copper nanoparticles with medicinal plants extracts: from extraction methods to applications. Micromachines 14, 1882. 10.3390/mi14101882 37893319 PMC10609153

[B15] Antunes Filho S. Dos Santos M. S. Dos Santos O. A. L. Backx B. P. Soran M.-L. Opriş O. (2023). Biosynthesis of nanoparticles using plant extracts and essential oils. Molecules 28, 3060. 10.3390/molecules28073060 37049821 PMC10095647

[B16] Atri A. Echabaane M. Bouzidi A. Harabi I. Soucase B. M. Chaâbane R. B. (2023). Green synthesis of copper oxide nanoparticles using Ephedra Alata plant extract and a study of their antifungal, antibacterial activity and photocatalytic performance under sunlight. Heliyon 9, e13484. 10.1016/j.heliyon.2023.e13484 36816263 PMC9929317

[B17] Auwal M. S. Saka S. Mairiga I. A. Sanda K. A. Shuaibu A. Ibrahim A. (2014). Preliminary phytochemical and elemental analysis of aqueous and fractionated pod extracts of Acacia nilotica (Thorn mimosa). Veterinary research forum: an international quarterly journal. Vet. Res. Forum 5, 95–100. 25568701 PMC4279630

[B18] Awala S. I. Oyetayo V. O. (2015). The phytochemical and antimicrobial properties of the extracts obtained from trametes elegans collected from osengere in ibadan, Nigeria. Jordan J. Biol. Sci. 8, 289–299. 10.12816/0027065

[B20] Baliyan S. Mukherjee R. Priyadarshini A. Vibhuti A. Gupta A. Pandey R. P. (2022). Determination of antioxidants by DPPH radical scavenging activity and quantitative phytochemical analysis of Ficus religiosa. Molecules 27, 1326. 10.3390/molecules27041326 35209118 PMC8878429

[B21] Barhoum A. García-Betancourt M. L. (2018). Physicochemical characterization of nanomaterials: size, morphology, optical, magnetic, and electrical properties. *Emerging applications of nanoparticles and architecture nanostructures* . Elsevier.

[B22] Benabderrahim M. A. Yahia Y. Bettaieb I. Elfalleh W. Nagaz K. (2019). Antioxidant activity and phenolic profile of a collection of medicinal plants from Tunisian arid and Saharan regions. Industrial Crops Prod. 138, 111427. 10.1016/j.indcrop.2019.05.076

[B23] Ben Amor I. Hemmami H. Laouini S. E. Mahboub M. S. Barhoum A. (2022). Sol-gel synthesis of ZnO nanoparticles using different chitosan sources: effects on antibacterial activity and photocatalytic degradation of AZO dye. Catalysts 12, 1611. 10.3390/catal12121611

[B24] Bouaziz A. Abdalla S. Baghiani A. Charef N. (2015). Phytochemical analysis, hypotensive effect and antioxidant properties of Myrtus communis L. growing in Algeria. Asian Pac. J. Trop. Biomed. 5, 19–28. 10.1016/s2221-1691(15)30165-9

[B25] Bouzid M. Lamine A. B. Chaâbane R. B. (2023). Biogenic of CuO nanoparticles using the plant extract Ephedra Alata applied to the removal of methylene blue from wastewater, and its statistical physics analysis. Res. Square. 10.21203/rs.3.rs-2710235/v1

[B26] Chen J. Xu F. Zhang Q. Li S. (2021). N-doped MoS2-nanoflowers as peroxidase-like nanozymes for total antioxidant capacity assay. Anal. Chim. Acta 1180, 338740. 10.1016/j.aca.2021.338740 34538313

[B27] Chen M. He X. Sun H. Sun Y. Li L. Zhu J. (2022). Phytochemical analysis, UPLC-ESI-Orbitrap-MS analysis, biological activity, and toxicity of extracts from Tripleurospermum limosum (Maxim.) Pobed. Arabian J. Chem. 15, 103797. 10.1016/j.arabjc.2022.103797

[B28] Danciu C. Muntean D. Alexa E. Farcas C. Oprean C. Zupko I. (2019). Phytochemical characterization and evaluation of the antimicrobial, antiproliferative and pro-apoptotic potential of Ephedra alata Decne. hydroalcoholic extract against the MCF-7 breast cancer cell line. Molecules 24, 13. 10.3390/molecules24010013 PMC6337526 30577537

[B29] Dappula S. S. Kandrakonda Y. R. Shaik J. B. Mothukuru S. L. Lebaka V. R. Mannarapu M. (2023). Biosynthesis of zinc oxide nanoparticles using aqueous extract of Andrographis alata: characterization, optimization and assessment of their antibacterial, antioxidant, antidiabetic and anti-Alzheimer's properties. J. Mol. Struct. 1273, 134264. 10.1016/j.molstruc.2022.134264

[B30] Djaafar Z. Ridha O. M. (2014). Phytochemical study of selected medicinal plant, solanum Nigrum, the Algerian Desert. Int. Lett. Chem. Phys. Astronomy 1, 25–30. 10.18052/www.scipress.com/ilcpa.20.25

[B31] Edrah S. M. Aljenkawi A. Omeman A. Alafid F. (2016). Qualitative and quantities analysis of phytochemicals of various extract for Ephedra altissima from Libya. J. Med. Plants Stud. 4, 119–121.

[B32] Elhadef K. Smaoui S. Fourati M. Ben Hlima H. Chakchouk Mtibaa A. Sellem I. (2020). A review on worldwide Ephedra history and story: from fossils to natural products mass spectroscopy characterization and biopharmacotherapy potential. Evidence-Based Complementary Altern. Med. 2020, 1–22. 10.1155/2020/1540638 PMC7210547 32419789

[B33] Faisal S. Jan H. Shah S. A. Shah S. Khan A. Akbar M. T. (2021). Green synthesis of zinc oxide (ZnO) nanoparticles using aqueous fruit extracts of Myristica fragrans: their characterizations and biological and environmental applications. ACS omega 6, 9709–9722. 10.1021/acsomega.1c00310 33869951 PMC8047667

[B34] Gatou M.-A. Kontoliou K. Volla E. Karachalios K. Raptopoulos G. Paraskevopoulou P. (2023). Optimization of ZnO nanoparticles’ synthesis via precipitation method applying taguchi robust design. Catalysts 13, 1367. 10.3390/catal13101367

[B35] Gharbi A. H. Hemmami H. Laouini S. E. Amor I. B. Zeghoud S. Amor A. B. (2023). Green synthesis of ZnO@ SiO2 nanoparticles using Calligonum comosum L. extract: an efficient approach for organic pollutant degradation in wastewater. Biomass Convers. Biorefinery 2023, 1–12. 10.1007/s13399-023-05063-2

[B36] Ghasemi P. A. Momeni M. Bahmani M. (2013). Ethnobotanical study of medicinal plants used by Kurd tribe in Dehloran and Abdanan districts, Ilam province, Iran. Afr. J. Traditional, Complementary Altern. Med. 10, 368–385. 10.4314/ajtcam.v10i2.24 PMC3746586 24146463

[B37] González-Juárez D. E. Escobedo-Moratilla A. Flores J. Hidalgo-Figueroa S. Martínez-Tagüeña N. Morales-Jiménez J. (2020). A review of the Ephedra genus: distribution, ecology, ethnobotany, phytochemistry and pharmacological properties. Molecules 25, 3283. 10.3390/molecules25143283 32698308 PMC7397145

[B39] Ilham B. A. Hemmami H. Laouini S. E. Temam H. B. Zaoui H. Barhoum A. (2023). Biosynthesis MgO and ZnO nanoparticles using chitosan extracted from Pimelia Payraudi Latreille for antibacterial applications. World J. Microbiol. Biotechnol. 39, 19. 10.1007/s11274-022-03464-5 36409376

[B40] Irshad S. Salamat A. Anjum A. A. Sana S. Saleem R. S. Naheed A. (2018). Green tea leaves mediated ZnO nanoparticles and its antimicrobial activity. Cogent Chem. 4, 1469207. 10.1080/23312009.2018.1469207

[B41] Ishwarya R. Tamilmani G. Al-Ghanim K. A. Govindarajan M. Nicoletti M. Vaseeharan B. (2023). Biosynthesis of zinc oxide nanoparticles from molted feathers of *Pavo cristatus* and their antibiofilm and anticancer activities. Green Process. Synthesis 12, 20230090. 10.1515/gps-2023-0090

[B42] Jan H. Shah M. Usman H. Khan M. A. Zia M. Hano C. (2020). Biogenic synthesis and characterization of antimicrobial and antiparasitic zinc oxide (ZnO) nanoparticles using aqueous extracts of the Himalayan Columbine (Aquilegia pubiflora). Front. Mater. 7, 249. 10.3389/fmats.2020.00249

[B43] Jaradat N. Dacca H. Hawash M. Abualhasan M. N. (2021). Ephedra alata fruit extracts: phytochemical screening, anti-proliferative activity and inhibition of DPPH, α-amylase, α-glucosidase, and lipase enzymes. BMC Chem. 15, 41–13. 34174945 10.1186/s13065-021-00768-9 PMC8235566

[B44] Jaradat N. Hussen F. Al Ali A. (2015). Preliminary phytochemical screening, quantitative estimation of total flavonoids, total phenols and antioxidant activity of Ephedra alata Decne. J. Mater. Environ. Sci. 6, 1771–1778. 10.1186/s13065-021-00768-9

[B45] Kalaimurugan D. Lalitha K. Durairaj K. Sivasankar P. Park S. Nithya K. (2022). Biogenic synthesis of ZnO nanoparticles mediated from Borassus flabellifer (Linn): antioxidant, antimicrobial activity against clinical pathogens, and photocatalytic degradation activity with molecular modeling. Environ. Sci. Pollut. Res. 29, 86308–86319. 10.1007/s11356-021-18074-1 35040048

[B46] Kancherla N. Dhakshinamoothi A. Chitra K. Komaram R. B. (2019). Preliminary analysis of phytoconstituents and evaluation of anthelminthic property of cayratia auriculata (*in vitro*). Maedica 14, 350–356. 10.26574/maedica.2019.14.4.350 32153665 PMC7035446

[B47] Khan M. Ware P. Shimpi N. (2021). Synthesis of ZnO nanoparticles using peels of Passiflora foetida and study of its activity as an efficient catalyst for the degradation of hazardous organic dye. SN Appl. Sci. 3, 528–617. 10.1007/s42452-021-04436-4

[B48] Kittana N. Abu-Rass H. Sabra R. Manasra L. Hanany H. Jaradat N. (2017). Topical aqueous extract of Ephedra alata can improve wound healing in an animal model. Chin. J. Traumatology 20, 108–113. 10.1016/j.cjtee.2016.10.004 PMC5392709 28209447

[B49] Kostova I. Bhatia S. Grigorov P. Balkansky S. S Parmar V. K Prasad A. (2011). Coumarins as antioxidants. Curr. Med. Chem. 18, 3929–3951. 10.2174/092986711803414395 21824098

[B50] Kulkarni D. Sherkar R. Shirsathe C. Sonwane R. Varpe N. Shelke S. (2023). Biofabrication of nanoparticles: sources, synthesis, and biomedical applications. Front. Bioeng. Biotechnol. 11, 1159193. 10.3389/fbioe.2023.1159193 37200842 PMC10185809

[B51] Kumar P. V. Shameem U. Kollu P. Kalyani R. Pammi S. (2015). Green synthesis of copper oxide nanoparticles using Aloe vera leaf extract and its antibacterial activity against fish bacterial pathogens. BioNanoScience 5, 135–139. 10.1007/s12668-015-0171-z

[B52] Lawag I. L. Nolden E. S. Schaper A. A. Lim L. Y. Locher C. (2023). A modified folin-ciocalteu assay for the determination of total phenolics content in honey. Appl. Sci. 13, 2135. 10.3390/app13042135

[B53] Mageshwari K. Mali S. S. Sathyamoorthy R. Patil P. S. (2013). Template-free synthesis of MgO nanoparticles for effective photocatalytic applications. Powder Technol. 249, 456–462. 10.1016/j.powtec.2013.09.016

[B54] Mahlaule-Glory L. M. Hintsho-Mbita N. C. (2022). Green derived zinc oxide (ZnO) for the degradation of dyes from wastewater and their antimicrobial activity: a review. Catalysts 12, 833. 10.3390/catal12080833

[B55] Mousavi S. M. Hashemi S. A. Ghasemi Y. Atapour A. Amani A. M. Savar Dashtaki A. (2018). Green synthesis of silver nanoparticles toward bio and medical applications: review study. Artif. cells, nanomedicine, Biotechnol. 46, 855–872. 10.1080/21691401.2018.1517769 30328732

[B56] Nguyen N. T. T. Nguyen L. M. Nguyen T. T. T. Nguyen N. H. Nguyen D. H. Nguyen D. T. C. (2023). Green synthesis of ZnFe2O4@ ZnO nanocomposites using Chrysanthemum spp. floral waste for photocatalytic dye degradation. J. Environ. Manag. 326, 116746. 10.1016/j.jenvman.2022.116746 36399883

[B57] Omran A. M. (2023). Characterization of green route synthesized zinc oxide nanoparticles using Cyperus rotundus rhizome extract: antioxidant, antibacterial, anticancer and photocatalytic potential. J. Drug Deliv. Sci. Technol. 79, 104000. 10.1016/j.jddst.2022.104000

[B58] Osuntokun J. Onwudiwe D. C. Ebenso E. E. (2019). Green synthesis of ZnO nanoparticles using aqueous *Brassica oleracea* L. var. italica and the photocatalytic activity. Green Chem. Lett. Rev. 12, 444–457. 10.1080/17518253.2019.1687761

[B60] Rabecca R. Doss A. Kensa V. M. Iswarya S. Mukeshbabu N. Pole R. P. (2022). Facile synthesis of zinc oxide nanoparticle using algal extract and their antibacterial potential. Biomass Convers. Biorefinery 2022, 1–12. 10.1007/s13399-022-03275-6

[B61] Radulescu D.-M. Surdu V.-A. Ficai A. Ficai D. Grumezescu A.-M. Andronescu E. (2023). Green synthesis of metal and metal oxide nanoparticles: a review of the principles and biomedical applications. Int. J. Mol. Sci. 24, 15397. 10.3390/ijms242015397 37895077 PMC10607471

[B62] Rahman F. Majed Patwary M. A. Bakar Siddique M. A. Bashar M. S. Haque M. A. Akter B. (2022). Green synthesis of zinc oxide nanoparticles using Cocos nucifera leaf extract: characterization, antimicrobial, antioxidant and photocatalytic activity. R. Soc. Open Sci. 9, 220858. 10.1098/rsos.220858 36425517 PMC9682308

[B63] Rajkumar K. S. Arun S. Babu M. D. Balaji P. Sivasubramanian S. Vignesh V. (2019). Facile biofabrication, characterization, evaluation of photocatalytic, antipathogenic activity and *in vitro* cytotoxicity of zinc oxide nanoparticles. Biocatal. Agric. Biotechnol. 22, 101436. 10.1016/j.bcab.2019.101436

[B64] Rasool A. Kiran S. Gulzar T. Abrar S. Ghaffar A. Shahid M. (2023). Biogenic synthesis and characterization of ZnO nanoparticles for degradation of synthetic dyes: a sustainable environmental cleaner approach. J. Clean. Prod. 398, 136616. 10.1016/j.jclepro.2023.136616

[B65] Rathnasamy R. Thangasamy P. Thangamuthu R. Sampath S. Alagan V. (2017). Green synthesis of ZnO nanoparticles using Carica papaya leaf extracts for photocatalytic and photovoltaic applications. J. Mater. Sci. Mater. Electron. 28, 10374–10381. 10.1007/s10854-017-6807-8

[B66] Sadiq H. Sher F. Sehar S. Lima E. C. Zhang S. Iqbal H. M. (2021). Green synthesis of ZnO nanoparticles from Syzygium Cumini leaves extract with robust photocatalysis applications. J. Mol. Liq. 335, 116567. 10.1016/j.molliq.2021.116567

[B67] Saidi S. A. Al-Shaikh T. M. Alghamdi O. A. Hamden K. (2022). Ephedra alata subsp. alenda (Ephedraceae) leaf extracts: phytochemical screening, anti-diabetic, anti-obesity and anti-toxic activities on diabetic-induced liver-kidney-testes toxicities and inhibition of α-amylase and lipase enzymes. Heliyon 8, e11954. 10.1016/j.heliyon.2022.e11954 36478797 PMC9720601

[B68] Saied E. Salem S. S. Al-Askar A. A. Elkady F. M. Arishi A. A. Hashem A. H. (2022). Mycosynthesis of hematite (α-Fe2O3) nanoparticles using Aspergillus Niger and their antimicrobial and photocatalytic activities. Bioengineering 9, 397. 10.3390/bioengineering9080397 36004922 PMC9404788

[B69] Saleem A. Saleem M. Akhtar M. F. Ashraf Baig M. M. F. Rasul A. (2020). HPLC analysis, cytotoxicity, and safety study of Moringa oleifera Lam.(wild type) leaf extract. J. Food Biochem. 44, e13400. 10.1111/jfbc.13400 32729119

[B70] Salman H. A. Yaakop A. S. Al-Mustafa A. Tarawneh K. Aladaileh S. Al-Rimawi F. (2021). The dual impact of Jordanian Ephedra alte for inhibiting pepsin and treating microbial infections. Saudi J. Biol. Sci. 28, 6245–6253. 10.1016/j.sjbs.2021.06.090 34764751 PMC8568995

[B71] Seghir B. B. Hima M. Moulatti F. Sahraoui I. Ben Amor I. Zeghoud S. (2023). Exploring the antibacterial potential of green-synthesized MgO and ZnO nanoparticles from two plant root extracts. Nanomaterials 13, 2425. 10.3390/nano13172425 37686933 PMC10489724

[B72] Senthilraja A. Subash B. Krishnakumar B. Rajamanickam D. Swaminathan M. Shanthi M. (2014). Synthesis, characterization and catalytic activity of co-doped Ag–Au–ZnO for MB dye degradation under UV-A light. Mater. Sci. Semicond. Process. 22, 83–91. 10.1016/j.mssp.2014.02.011

[B73] Shraim A. M. Ahmed T. A. Rahman M. M. Hijji Y. M. (2021). Determination of total flavonoid content by aluminum chloride assay: a critical evaluation. LWT 150, 111932. 10.1016/j.lwt.2021.111932

[B74] Singh H. Desimone M. F. Pandya S. Jasani S. George N. Adnan M. (2023). Revisiting the green synthesis of nanoparticles: uncovering influences of plant extracts as reducing agents for enhanced synthesis efficiency and its biomedical applications. Int. J. Nanomedicine 18, 4727–4750. 10.2147/ijn.s419369 37621852 PMC10444627

[B75] Singh P. Singh H. Kim Y. J. Mathiyalagan R. Wang C. Yang D. C. (2016). Extracellular synthesis of silver and gold nanoparticles by Sporosarcina koreensis DC4 and their biological applications. Enzyme Microb. Technol. 86, 75–83. 10.1016/j.enzmictec.2016.02.005 26992796

[B76] Sohail M. F. Rehman M. Hussain S. Z. Huma Z.-E. Shahnaz G. Qureshi O. S. (2020). Green synthesis of zinc oxide nanoparticles by Neem extract as multi-facet therapeutic agents. J. Drug Deliv. Sci. Technol. 59, 101911. 10.1016/j.jddst.2020.101911

[B77] Soumaya B. Yosra E. Rim B. M. Sarra D. Sawsen S. Sarra B. (2020). Preliminary phytochemical analysis, antioxidant, anti-inflammatory and anticancer activities of two Tunisian Ephedra species: ephedra alata and Ephedra fragilis. South Afr. J. Bot. 135, 421–428. 10.1016/j.sajb.2020.09.033

[B78] Suresh J. Pradheesh G. Alexramani V. Sundrarajan M. Hong S. I. (2018). Green synthesis and characterization of zinc oxide nanoparticle using insulin plant (Costus pictus D. Don) and investigation of its antimicrobial as well as anticancer activities. Adv. Nat. Sci. Nanosci. Nanotechnol. 9, 015008. 10.1088/2043-6254/aaa6f1

[B79] Thaipong K. Boonprakob U. Crosby K. Cisneros-Zevallos L. Byrne D. H. (2006). Comparison of ABTS, DPPH, FRAP, and ORAC assays for estimating antioxidant activity from guava fruit extracts. J. food Compos. analysis 19, 669–675. 10.1016/j.jfca.2006.01.003

[B80] Thi T. U. D. Nguyen T. T. Thi Y. D. Thi K. H. T. Phan B. T. Pham K. N. (2020). Green synthesis of ZnO nanoparticles using orange fruit peel extract for antibacterial activities. RSC Adv. 10, 23899–23907. 10.1039/d0ra04926c 35517333 PMC9055061

[B81] Tungmunnithum D. Thongboonyou A. Pholboon A. Yangsabai A. (2018). Flavonoids and other phenolic compounds from medicinal plants for pharmaceutical and medical aspects: an overview. Medicines 5, 93. 10.3390/medicines5030093 30149600 PMC6165118

[B82] Vaseem M. Lee K.-M. Shin J.-K. Hahn Y.-B. (2012). Synthesis of ZnO nanoparticles and their ink-jetting behavior. J. Nanosci. Nanotechnol. 12, 2380–2386. 10.1166/jnn.2012.5693 22755062

[B83] Vijayakumar S. Mahadevan S. Arulmozhi P. Sriram S. Praseetha P. (2018). Green synthesis of zinc oxide nanoparticles using Atalantia monophylla leaf extracts: characterization and antimicrobial analysis. Mater. Sci. Semicond. Process. 82, 39–45. 10.1016/j.mssp.2018.03.017

[B84] Xue Y. Bin Ismail A. J. Lansing M. G. Bin Mohd Hayati M. F. (2023). Novel green synthesis of zinc oxide nanoparticles using Salvia rosmarinus extract for treatment of human lung cancer. Open Chem. 21, 20230113. 10.1515/chem-2023-0113

[B85] Zeghoud S. Rebiai A. Hemmami H. Ben Seghir B. Elboughdiri N. Ghareba S. (2021). ATR–FTIR spectroscopy, HPLC chromatography, and multivariate analysis for controlling bee pollen quality in some Algerian regions. ACS omega 6, 4878–4887. 10.1021/acsomega.0c05816 33644595 PMC7905949

[B86] Ziani B. E. Heleno S. A. Bachari K. Dias M. I. Alves M. J. Barros L. (2019). Phenolic compounds characterization by LC-DAD-ESI/MSn and bioactive properties of Thymus algeriensis Boiss. and Reut. and Ephedra alata Decne. Food Res. Int. 116, 312–319. 10.1016/j.foodres.2018.08.041 30716951

